# ”Living with heart failure” –patients’ and nurses’ experience using an online support program via a national e-health platform – a feasibility study

**DOI:** 10.1186/s12912-026-04370-z

**Published:** 2026-02-04

**Authors:** Emma Säfström, Anna Strömberg, Marie Lundberg, Patric Karlström, Charlotta Lans, Carina Svenlin, Maria Liljeroos

**Affiliations:** 1 Department of Health, Medicine and Caring Sciences, Linköping University, Linköping, Sweden; 2 Centre for Clinical Research Sormland, Uppsala University, Eskilstuna, Sweden; 3 Department of Cardiology, Linköping University Hospital, Linköping, Sweden; 4 Department of Internal Medicine, Ryhov County Hospital, Jönköping, Sweden; 5 Department of Physiotherapy, Region Kalmar County, Kalmar County Hospital, Kalmar, Sweden; 6 Department of Cardiology, Vrinnevi Hospital, Norrköping, Sweden

**Keywords:** Heart failure, Elderly, E-health intervention, Online support program

## Abstract

**Background:**

Heart failure is increasingly common among the elderly, yet many are not referred to specialized care. To better support this group, a co-designed e-health program for patients with heart failure was developed and implemented on Sweden’s national health platform, 1177.

**Aim:**

To investigate patients and heart failure nurses’ perception of the feasibility of an online support program “Living with Heart Failure”.

**Methods:**

A feasibility study including interviews with patients and nurses. Four heart failure nurses recruited patients from outpatient clinics in two Swedish regions through convenience sampling. Patients used the program for 12 weeks before being interviewed about its usability, design, features, and content. Nurses were interviewed in a group setting using similar questions. All interviews were analysed using deductive content analysis, and quantitative data described patient characteristics and program use.

**Results:**

Eleven patients were included, comprising five women and six men, aged 47–84 years (mean 66.8). All had used the program at least once and found the design clear and navigation intuitive. The module content was informative and written in accessible language. They appreciated the variety of content formats. Nurses found the program easy to use and liked the layout but identified limited time as a key barrier to implementation.

**Conclusion:**

Patients and nurses found the support program feasible regarding acceptability, demand, and practicality. The study also indicated a need for future adjustments and showed that the relevance of the program is not restricted to individuals with newly diagnosed heart failure.

**Trial registration:**

Clicicaltrials.org 2023-04289-01 date of submission for registration 10/11/2025.

**Supplementary Information:**

The online version contains supplementary material available at 10.1186/s12912-026-04370-z.

## Introduction

Heart failure is a growing global health challenge which affects the adult population in general, while remaining more prevalent among elderly individuals, with prevalence reaching 10% in those over 80 years [1, 2]. Although symptoms can be alleviated through guideline-directed medical therapy (GDMT) and patient self-care [3], care models must evolve to integrate digital innovations that support treatment optimization and patient engagement [4]. E-health interventions enable patients to monitor their health, recognize early symptoms, and collaborate with healthcare professionals [5, 6]. Patients with heart failure generally have a positive attitude toward e-health, and digital health literacy has been shown to improve self-management and symptom control [6–8].

Healthcare professionals acknowledge the potential of e-health to improve access and continuity of care but emphasize the need for adaptation in workflows and professional roles [9]. Nurses play a key role in promoting e-health among patients, as their recommendations strongly influence patient acceptance [6]. In Sweden, a co-designed online support program, “Living with Heart Failure”, was developed collaboratively with patients and nurses and made available through the national health portal 1177. Before large-scale implementation, its feasibility in clinical practice must be assessed [10, 11].

The present study aimed to investigate patients’ and heart failure nurses’ perceptions of the feasibility of the online support program “Living with Heart Failure”, focusing on acceptability, demand, implementation, practicality, and integration, designed for patients newly diagnosed with heart failure.

## Background

Heart failure is a global health challenge that is escalating as the prevalence of patients living with heart failure is increasing [1]. The prevalence of heart failure in the 70–79 age group is 7%, and in persons older than 80 years, it is 10% [2]. It is a complex progressive chronic condition and continuous, person-centered support from healthcare professionals is important to reduce symptoms, improving quality of life and prognosis [3]. Further, care models and clinical pathways for heart failure must evolve and adapt to recent innovations in technology to manage the increasing burden of the condition. Digital health tools can help optimize GDMT prescriptions and support patient self-care [4]. Digital health is an umbrella term that includes e-health, which has been defined as the “*use of information and communications technologies in support of health and health-related fields, including health-care services, health surveillance, health literature, and health education, knowledge and research*” [5].

E-Health interventions offer patients the opportunity to collaborate with healthcare professionals in monitoring and managing their conditions. They encourage patients to adopt healthy lifestyles while also enabling the monitoring of key health indicators, early symptom recognition, and the identification of signs of deterioration [6]. Although patients with heart failure are often older, they generally have a positive attitude towards e-health, and over 70% could see themselves using such an intervention to manage their condition. Additionally, their attitude toward e-health was not affected by age, heart failure level, employment, or marital status [6]. In patients with heart failure, a recent study found that digital health literacy can reduce the severity of symptoms by enhancing individuals’ health perceptions and behaviours [7]. Additionally, e-health has been shown to improve physical capacity through rehabilitation via an app [8].

Most healthcare professionals view technology as a means to enhance equal health and access to care (World Health Organization, 2021), but there is less consensus on how to implement it into daily clinical practice. An observational study revealed that healthcare providers consider technology a new way to interact with patients. Although pragmatic, they emphasized that this requires not only learning new skills but also rethinking current routines and professional roles [9]. Healthcare workers, especially nurses, play a crucial role in promoting e-health among patients. Patients with heart failure indicated that they would be more likely to consider using an e-Health intervention if it were recommended by a heart failure nurse [6].

One example of an e-health intervention for patients with heart failure is an online support program, “Living with heart failure”, recently developed through a co-design project involving heart failure nurses and patients. The key concept of the program includes essential information on heart failure, diagnosis, treatment and how to manage self-care and daily life. The information is provided though videos, lectures, interviews as well as text and pictures. It also includes the possibility to send messages to the heart failure nurse and report blood pressure and weight. The support program is available through the Swedish national online health portal, 1177. The portal provides information on health conditions and healthcare services and allows citizens to log in using electronic identification. Secure access enables users to view medical records, test results, prescribed medications, and scheduled healthcare appointments.

Although some studies indicate a positive impact of e-health and telemonitoring [12], a review of digital solutions for heart failure reveals that there are lessons to be learned: the integration of e-health into routine clinical practice remains limited, and it is crucial to consider the human factors involved in its implementation. Both patients and healthcare professionals require support. e-Health should be introduced through a co-design and co-implementation process with patients and healthcare professionals [10]. Before implementing the intervention on a large scale, it is advisable to assess its feasibility [11].

## Aim

To investigate patients and heart failure nurses’ perception of the feasibility of the online support program “Living with Heart Failure” regarding acceptability, demand, implementation, practicality, integration, and designed for patients newly diagnosed with heart failure.

## Methods

### Design and settings

This feasibility study used both qualitative and quantitative methods as recommended [11, 13]. Feasibility studies are particularly important for complex interventions that involve multiple components or levels of settings. When evaluating complex interventions, some of the core elements is to identify key refinements such as fine tuning or making changes in the intervention. The results can inform decisions on how to proceed with the next stage of evaluation. A feasibility study can assess various aspects of the intervention itself, such as its content, delivery, acceptability, or adherence [14]. A framework for designing feasibility studies has been developed by Bowen and colleagues [11], encompassing eight key areas of feasibility that should be considered. The proposed areas are acceptability, demand, implementation, practicality, adaptation, integration, expansion, and limited efficacy. Each area can be used to answer the question of whether an intervention can work, does work, or will work.

Bowen et al.’s framework is intended to be applied flexibly, with the selection of domains guided by the specific aims and developmental stage of the intervention. The scope of this study is to investigate whether an online support program will work in real-life settings by examining five of the areas (acceptability, demand, implementation, practicality, and integration). This is the first step before further evaluating the implementation and effectiveness of the support program. Data was collected between October 2024 and December 2024.

### Participants and recruitment

Nurses working with heart failure patients were eligible for inclusion. There were no exclusion criteria. Nurses were recruited by the research group from outpatient heart failure clinics in hospital and primary care settings in two regions in southeastern Sweden using convenience sampling. These nurses subsequently recruited patients from hospital-based heart failure and primary care clinics within the same regions, also using convenience sampling. Patients eligible for inclusion were those over 18 years of age and who were diagnosed with heart failure. To use the program, patients were required to have access to a digital device, such as a computer, tablet, or smartphone. They also needed to have e-identification to log in to 1177.se, as the program is personalized.

Exclusion criteria were patients with cognitive impairment, severe mental illness or non-Swedish speakers. Additionally, patients with an expected short life expectancy (< 6 months) were excluded.

All participants received verbal and written information, and informed consent was obtained at baseline. The Consolidated Criteria for Reporting Qualitative Research (COREQ) were followed to enhance reporting, see Appendix 1 in the Supplementary Data section.

### The intervention

The support program *Living with Heart Failure* was developed through a co-design process involving patients and heart failure nurses. During the process, several workshops were conducted to identify end users’ preferences and needs. Data from the co-design workshops were integrated into mind maps for each stage of the patient journey. Nurses then accessed the program, and their experiences were evaluated through individual and group online interviews, resulting in minor revisions. Additionally, three patients participated in brief online testing of the draft program, with feedback collected via interviews. The final content was developed collaboratively by the research group and content experts and included text, images, interviews, and lectures, with revisions made as needed to ensure readability.

**Fig. 1:**
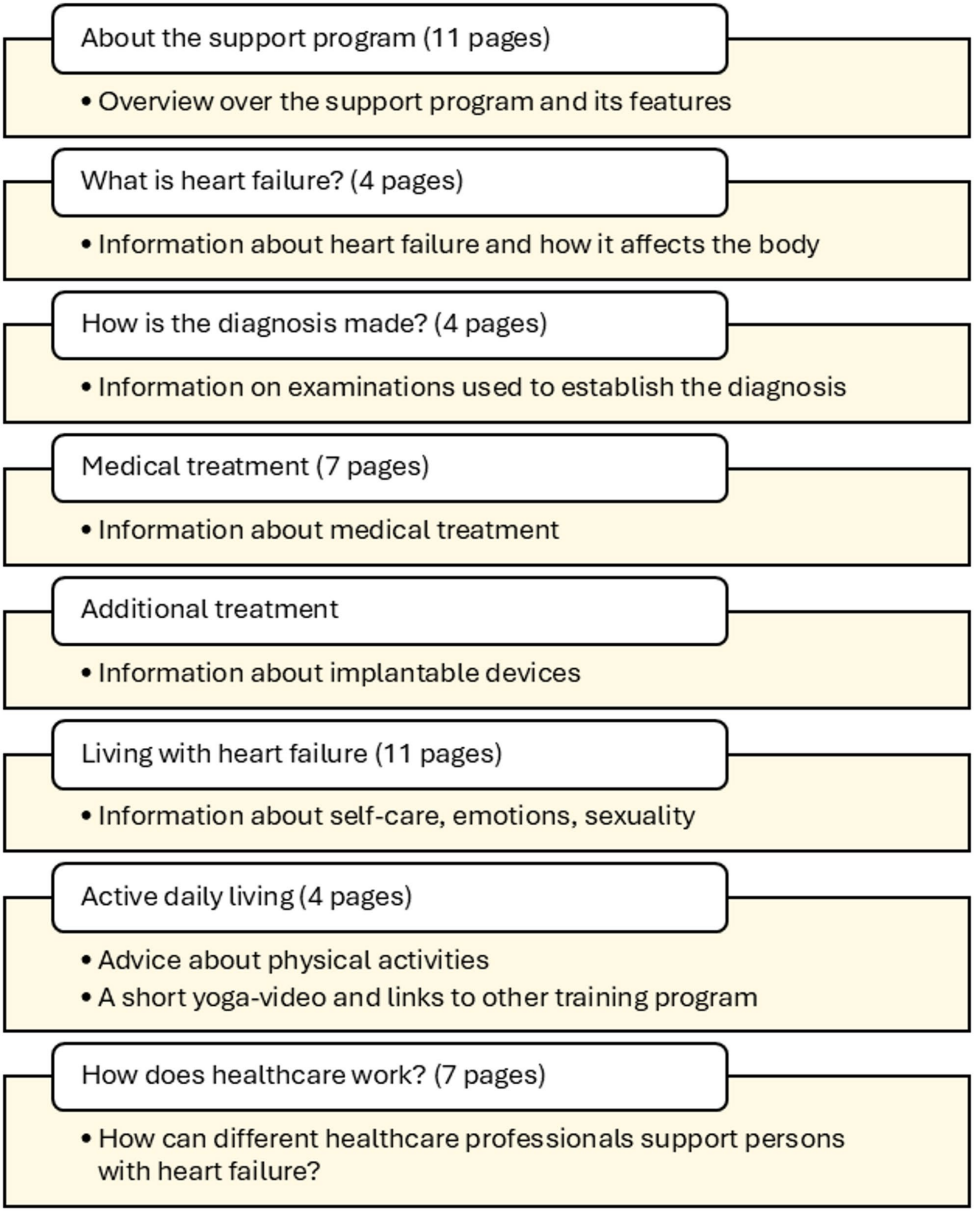
Modules in the support program

Initially intended for newly diagnosed patients, it comprises eight modules covering topics such as understanding heart failure, diagnostic procedures, medical treatment, self-care, physical activity, and the roles of healthcare professionals (Fig. 1). In total, the program includes 49 web pages featuring texts, recorded lectures, interviews, images, and patient stories. Additional features include secure messaging between patients and nurses, options to report blood pressure and weight—with alerts for abnormal values—a planning tool for appointments, and a quiz on heart failure management. Nurses provide patients with access to the program, which can be revisited freely and navigated in any order.

### Data collection and procedures

Data collection was conducted continuously after the included participants had access to the program for 12 weeks. There is no established standard regarding the duration of feasibility studies; rather, the timeframe should be guided by the study objectives and the ability to address key feasibility outcomes. Three-month feasibility periods are commonly used in clinical and intervention research to assess acceptability, usability, and procedural feasibility prior to larger-scale evaluation. Thereafter, a phone interview was scheduled with the participant which were audiotaped and transcribed verbatim. The interviews were performed by one of the authors (CS). The interview was based on a semi-structured interview guide, with open-ended and probing questions (Appendix 2 in the Supplementary Data section). The questions were developed based on the areas described in the implementation of a feasibility study (Bowen et al., 2009). The research questions aimed to determine whether the patient had used the program and, if so, how the participants perceived the program’s usability, design, features, and content. Example questions included: “What was your first impression of the program?“, “Was there any part of the program that was particularly important to you?“, “Would you recommend the support program to others with heart failure?“. To further develop the responses, probing questions were asked. The participants who had not used the support program were asked the reasons for not using the program.

The interview duration varied, with the shortest interview lasting 8 min and the longest lasting 22 min, median 14, 06 min.

Quantitative data were collected to describe the patients’ characteristics and actual use of the support program. Demographic data were collected via a study-specific questionnaire. Data regarding the use of the support program was extracted from the 1177. It was only possible to extract data on which module the patients have opened, not the frequency or duration.

The heart failure nurses were interviewed in a semi-structured online focus-group interview. A modified version of the patient interview guide was used. It included additional questions about prior experience with similar programs and a question regarding the integration of the support program in a clinical setting. The interview lasted for 50 min. Table 1 describe the data collection and analysis methods.

**Table 1:** Overview of the aspects of feasibility examined, as well as methods for data collection and analysis

Area of focus for feasibility	Aspects of feasibility	Methods for data collection	Methods for data analysis
*Acceptability* To what extent is the support program judged suitable, satisfying, or attractive to patients?	Perceived appropriateness	Interviews with patients and heart failure nurses.	Deductive content analyses
	Satisfaction with the online support program		
	Intent to continue to use the online support program		
*Demand* To what extent is the online support program likely to be used?	Expressed interest or intention to use the online support program		
	Perceived demand		
	Actual use of the support program	Data on which modules and functions of the online support program the patients used.	Descriptive statistics
*Implementation* To what extent can the online support program be successfully delivered to the intended participants?	Successful or unsuccessful use of the program as intended		
		Interviews with patients and heart failure nurses.	Deductive content analyses
	Resource requirements to support implementation (e.g. staff support, technical assistance)		
*Practicality* To what extent can the intervention be carried out with intended participants using existing means, resources, and circumstances?	Feasibility of using the program under real-world conditions		
	Perceived usability and burden for participants		
*Integration* To what extent can the intervention be integrated into an existing system?	Perceived fit of the program within everyday life and care routines Acceptability of continued use beyond the study period	Interviews with heart failure nurses about their perceived fit of the online support program within the clinical practice	

### Analysis

The qualitative data from the interviews was analysed using direct deductive content analysis as described by Elo & Kyngäs [15]. The feasibility of the support program was studied according to the description of various aspects of feasibility as described by Bowen et al. [11].

First, all interviews were transcribed verbatim. To get a sense of the whole, the transcripts were read several times by authors ML and ES who also did the initial coding. In the next step, the feasibility areas—acceptability, demand, implementation, practicality, and integration—were used as predetermined concepts for coding the text. Coding of the transcripts was done by highlighting text describing the feasibility areas. Text describing the five areas were made into categories. According to Bowen et al. each feasibility area consists of various aspects. In the next step of the coding, text describing the feasibility aspects were made into subcategories.

Interview duration was documented, and Table 1 outlines data collection and analysis procedures. Categories and subcategories were discussed among all authors. Results are presented according to Bowen et al.’s areas: acceptability, demand, implementation, and practicality.

Confirmability was supported through detailed documentation of procedures and the use of an established framework. Quantitative data on patient characteristics and program use were analyzed descriptively to complement qualitative findings. The study setting, participant characteristics, and the support program are described in detail to enable assessment of transferability. All participating nurses were experienced in heart failure care.

## Results

### Participants’ characteristics

A total of 11 patients were included in the study, comprising five women and six men, with ages ranging from 47 to 84 years. They had heart failure for two to 47 months. Patients’ demographic and clinical characteristics is outlined in Table 2. Most of them were familiar with using a smartphone or a computer as well as the 1177 platform. During the scheduling process for upcoming interviews, two patients chose to decline the interview. Therefore, a total of nine patients were included in the content analysis.

**Table 2:** Patients’ demographic and clinical characteristics (*n* = 11)

Variable		
Age, mean (SD)	66.8	(12.5)
Living with spouse, n (%)	9	(82)
Level of education, n (%)		
Primary school	2	(18)
Secondary school	3	(33)
University or equivalent	4	(44)
(Missing)	2	(18)
Months since diagnosed with HF, median (IQR)	5	(3–12)
Ethology		
Hypertension	2	(18)
Cardiomyopathy	2	(18)
Arrhythmia	2	(18)
Unknown	4	(36)
(Missing)	1	(9)
NYHA-class		
I	2	(18)
II	8	(73)
III	0	(0)
IV	0	(0)
(Missing)	1	(9)
EF %, mean (SD)	32.5	(18.1)
Medical treatment, n (%) (1 missing on all variables)		
ACE or ARNI	7	(64)
BB	10	(90)
MRA	7	(64)
ARB	3	(27)
SGLT-2	9	(82)

Abbreviations: ACE; angiotensin converting enzyme, ARB; angiotensin II receptor blockers, ARNI; angiotensin receptor-neprilysin inhibitors, BB; beta blockers, EF; ejection fraction, HF; heart failure, IOR; interquartile range, MRA; mineralocorticoid receptor antagonist, NYHA; New York heart association, SGLT-2; sodium glucose cotransporter 2

Four heart failure nurses were included in the study. They were all women and worked in specialized hospital-based heart failure clinics (*n* = 3) as well as in primary healthcare centres (*n* = 1). All nurses except one had previous experience managing a support program in 1177.

### Acceptability

#### Perceived appropriateness and satisfaction with the online support program

Once logged in, most patients found that the program featured a clear layout and intuitive navigation. They found the support program appropriate, the modules informative and the text was written in a clear language they could understand. They also appreciated that the content was delivered in various formats.

No, I think the information has been good, simple, and clear. I usually judge it based on whether I understand it even without having any medical training, and I feel like I do — and that’s a good thing. (Patient)

The patients also found that the program could be appropriate for family members who wish for more information but not able to follow the patient to the hospital visits and get first-hand information.

The overall impression from the nurses was that the support program was easy to use and they liked the layout of the program.

It is a nice-looking program and it feels easy to use, clear in its design and nicely done. That was my first impression, it looks accessible, welcoming (Nurse)

The nurses found the scope of the support program appropriate and reported that it was easy to use. They specifically appreciated that the program was easy to get an overview of, that it contained relevant information without being overly extensive, and that it had an attractive layout and appealing images. They felt that it was beneficial for patients to have access to all the modules from the start, as this allows them to select the areas of information most relevant to them. Dividing the content into modules facilitates revisiting and rereading specific parts of the material. They also valued the ability to send and receive messages from patients.

Yes, it looks inviting and nice, it’s a bit easy to get an overview of, and it doesn’t take that long to go through everything. (Nurse)

No, I agree with that because I think the scope is very good. I think it mustn’t be too long because then you lose—lose the patients’ focus and interest somehow. But from having seen the whole way, so to speak, I think the final product still turned out nice. (Nurse)

They discussed the appropriate timing for introducing the program to patients, considering whether it should be offered at the time of diagnosis or after a certain period had elapsed. On one hand, patients receive a lot of information about medical treatment and self-care, and the support program could be especially helpful in providing this information. On the other hand, the patient may be overwhelmed by a new diagnosis and struggle to understand the information about the support program and how to use it.

#### Intent to continue to use the online support program

During the study, patients had access to the program for three months, but they found the period a bit too short. If the program should be implemented into clinical practice, they perceived that it would be helpful to have access at least for six months or more.

I think it’s great to have it for life, really. I mean, if you have heart failure, it’s a chronic condition that lasts your whole life, so it’s wonderful to be able to go back in and maybe… and I feel now, since it’s been a while since I last logged in, that tonight I’ll probably go in and check it out again. I think it’s important just to refresh my knowledge a bit and update myself so that I know even more. (Patient)

### Demand

#### Expressed interest or intention to use the support program

Some patients had used the possibility to report blood pressure and weight and found it helpful since they could consult the nurse if values changed.

That’s what I’ve been doing — logging in every day and entering things like my blood pressure and weight. (Patient)

Others perceived it as voluntary or felt that it had not been communicated by the nurse, and therefore not reported any measurements.

Only two participants answered the quiz, and some had not seen this feature.

The majority appreciated the message function which was an easy way to communicate with the nurse.

#### Perceived demand

Being diagnosed with heart failure can be a burdensome and chocking experience for some, often raising many questions. Access to a support program was described as positive, providing a place patients can find answers.

I think so, because you just feel so lost. Like—what is this? Am I going to die now? Is there anyone else who has this? What should I do? Can I go out? Can I exercise? Can I—oh no—and what should I even eat? I mean, there are like a hundred million questions that pop up. (Patient)

Since heart failure is a chronic condition, patients highlighted the relevance of having access to the program when needed even if they were diagnosed a long time ago.

If I reflect on my own journey, I would have really appreciated something like this — especially when I first got my diagnosis. At that time, some people might shut down emotionally, which is also a natural reaction. So maybe the material needs to stay available for a longer period, not just right at the start. But personally, I was very eager to get information as soon as I got my diagnosis and to read everything I could. Still, we’re all different, so some people might need a year to process it. That way, they can read from the beginning, return to it later, and maybe even retake the quiz — and hopefully get better results than last time. (Patient)

The nurses reflected on the perceived demand of the support program for newly diagnosed patients. The support program was developed with newly diagnosed patients in mind; however, the nurses expressed some concerns regarding the appropriate timing of introducing the support program to a newly diagnosed patient and also reflected that the support program might be of great value for a patient who had heart failure for some time, but is deteriorating. They also expressed concern that the support program might cause anxiety or unease because it reminds them that heart failure is a serious chronic condition.

That’s the risk you take with any kind of information really. It can do both good and harm, and we can’t, like, we can’t wrap the patients in cotton and not be honest and say that it’s serious. But at the same time, you can also be mindful of, just like you said, does this patient benefit from getting a full support program right now, or should we maybe wait a bit further along in the process? (Nurse)

#### Actual use of the support program

The quantitative evaluation of the implementation revealed that during the 12-week study period, only two patients accessed all eight modules in the support program. The module accessed by the most patients was the first module (“About the support program”). Six patients accessed the modules “What is heart failure,” “Medical treatment,” and “Living with heart failure” (Table 3).

The most used feature of the support program was the ability to send messages to the heart failure nurse, which all patients used. Four patients had reported blood pressure and weight on at least one occasion (Table 3).

**Table 3:** Overview of the number of patients that have accessed each module of the support program, and the features used

Name of the module	Number of patients accessed each module (*n* = 11)
About the support program	8
What is heart failure?	7
How do you get diagnosed with heart failure?	5
Medical treatment	6
Other treatment	3
To live with heart failure	6
Physical activity	5
How does the healthcare system work?	2
**Features**	
Sent at least one message to the heart failure nurse	9
Reported blood pressure and weight	4
Answered the Quiz	2

### Implementation

#### Success or failure in executing the implementation

As presented in Table 3, only a few patients had accessed all the modules, and half of the patients had only read half of the modules. One patient interpreted the information that blood pressure was to be reported daily, which made it feel overwhelming to use the program. For other patients, returning to the program had simply not taken place. The nurses reflected that they perhaps should have sent reminders to patients who had not logged on to the program after a few weeks.

#### Amount and type of resources needed to implement

Almost all patients managed to log in to the support program without help when using the written instructions provided by the 1177 platform. One patient received help from her husband the first time she logged in but could manage after that.

Well, at first I probably couldn’t, so I asked my husband, and he helped me — but now I think I actually can. (Patient)

The nurses discussed the type of support they could provide in a clinical setting to help patients implement the program. They believed that supporting patients when they first used the support program would facilitate its use and implementation. They suggested support in the form of showing and instructing patients on how to access the support program on 1177, introducing the program, and recommending which modules could be of interest to the patient to start with, based on their knowledge of the patient and prior discussions between the nurse and the patient. They, however, recognized that a possible barrier to supporting patients is the limited time available during clinical visits. They also discussed nudging as a support for implementation, whereby nurses should send welcome messages and reminders within the program to patients.

The nurses reflected that a key factor in facilitating the implementation of the support program is having sufficient time during patient visits to introduce and demonstrate the program.

### Practicality

#### The ability of participants to carry out intervention activities

Patients expressed an interest in the support program and had logged in at least once. One participant experienced a high workload during the study period and had only reviewed the program superficially at one single occasion. However, he expressed a positive attitude toward engaging with the program again once his workload had decreased.

Yes, I only went in at the beginning and looked through everything a bit, but then… well, to be honest, I run my own business and work around the clock. So, you know, I’m just happy when I feel well and can work. (Patient)

One participant noted the lack of a search function to help quickly find specific information or answers to a certain question and also that the text in the user guide was small and difficult to read.

Yes, I was able to access the program, but I can’t access the quick guide — I can open it, but I can’t read anything in it. (Patient)

The nurses could start using the support program after a brief introduction and found it easy to use. They did not experience any issues with logging in to the program or making it accessible to their patients.

..so it was nothing complicated and starting it and not, so to speak, adding patients, so there were no issues… (Nurse)

#### Factors affecting implementation ease or difficulty

A few participants had problems to find the program when they tried to log in for the first time. They found it to be so much information on the log in page of the platform and not intuitive where to find different programs. An icon highlighting the program would have been helpful.

The nurses identified several factors that might make the support program difficult to implement, including patients becoming overwhelmed with information when first log in to the program and forgetting they have access to it, as well as the layout of the 1177 webpage making it challenging for patients to locate the support program. They also noted some technical issues, such as one patient sending a message to the nurse about the text size in the support program, saying it was too small to read.

– Yes, I think so, now this is maybe a bit off from that question, but just this experience when you come into 1177.se, that there is very much information, and just like this thing that you should open the program or at least show visually that even if they maybe don’t remember it. (Nurse)

### Integration

#### Perceived fit with infrastructure/workflow or clinical setting

The support program had features that the nurses perceived as well-fitting within the infrastructure and workflow of the clinical setting, particularly the ability for patients and nurses to send messages to each other and the reporting of blood pressure.

For example, if patients have a short question, it is convenient to use the message function. However, in cases of questions regarding assessing symptoms or deterioration, the nurses still preferred to talk to the patient rather than write messages.

– Yes, like the echo, has the referral been sent or something, and then you can kind of feel that, well, maybe that’s not the most optimal way to use our phone consultation time. Something like that would have been really easy to respond to via 1177 [the message feature], like, but it is important for patients to know whether they’re supposed to do an echo or not, but maybe you don’t really need to go through the whole TeleQ thing [call queue] and wait for a call back and all that. (Nurse)

– Yeah, and I also think the threshold might be lower to send a message via 1177 than to call during a phone slot – just because you might feel that the question is kind of small, but you’d still like an answer to it. Or that you might get a flow going where, like, a patient who is unwell – maybe you’ve already talked on the phone – but then you can almost check in daily on how things are going just via the program. (Nurse)

Also, being able to see the patients’ blood pressure was perceived as convenient by the nurses. Especially during periods of adjusting the medical treatment to manage symptoms, but not on a day-to-day basis.

… you don’t really want to log in every day just to confirm that, okay, this person has reported this. But on the other hand, there might be patients who are maybe unstable and who’ve called because they’re really, really dizzy, and then you’ve decided, like, take your blood pressure for a few days and then we’ll see if it’s still at, like, systolic 86 – because then we’ll have to reduce your medication. Then I might call that patient after two days, but instead you could actually communicate via the messages, like, saying: “No, I’m not dizzy anymore and the blood pressure is a bit higher,” – yeah, then you can just let it go, kind of. (Nurse)

## Discussion

This study investigated the feasibility of the online support program “Living with Heart Failure” designed for patients newly diagnosed with heart failure. The demand for self-care technologies has markedly increased since the COVID-19 pandemic, further accelerating their adoption. Digital solutions offer a unique capacity for remote symptom monitoring and patient support [12, 16], while potentially improving resource utilization and decreasing the overall cost of care [17].

The findings suggest that the program had high acceptability, demand, and practicality by both patients and nurses.

Both patients and nurses found the support program acceptable both regarding content and layout, and the program was easy to use. These results are promising, as patients’ perceptions of eHealth interventions as easy to use play a key role in facilitating engagement with and sustained use of such programs [18]. One reason for the high acceptance could be that the program was developed using co-design, which also have been found in previous research [19]. A co-designed support programme has the capacity to be both accessible and meet end users’ needs and align with person-centred care. Although a recent systematic review reported effects on clinical outcomes, with improvements observed in medication adherence and self-care behaviours using mHealth-delivered education for people with heart failure [20], the high level of acceptance observed in the present study is encouraging. Given that acceptability and engagement are key prerequisites for the effectiveness of digital interventions, these findings suggest that the support program has the potential to support self-care.

Regarding demand, all patients logged in to the program at least once, but not all used the features in the program. Some reported seeing no need to enter measurements because they had not been instructed to do so. Others had not noticed the available features, suggesting that these may have been presented clearly within the program. A similar result was found in a recent study testing a mobile app, called Heart Failure-Smart Life [12]. Of the 36 participants in the intervention group, 66% reported that they had participated in the mobile app program regularly. Those results underscoring variability in engagement with digital interventions among patients.

Patients with relatives reported that unfortunately, their relatives’ ones did not show any direct interest in the program and its content. Receiving support from relatives has a significant role in maintaining motivation for self-care and providing practical assistance for patients with heart failure. Additionally, mental health is positively affected by receiving social support, which can further strengthen self-care behaviours [21].

Therefore, there is value in involving relatives when activating the support program. Hopefully, the support program can also increase the understanding among relatives of what a diagnosis of heart failure entails and what it means from a caregiver’s perspective.

There were also areas that challenged feasibility: Most of the participants encountered no difficulties as to logging in to the support program. However, some participants reported that it was difficult to locate the support program on the log in page of the platform. As the platform itself was beyond our control, we created a user guide providing patients with step-by-step instructions on how to locate the program. Challenges to the feasibility reported by the nurses was that introducing the support program would take time from the nurses, necessitating the de-prioritization of other tasks. However, they recognized that nurses may be particularly well positioned to initiate and support patients’ engagement with the support program. Patients likewise acknowledges that nurses have a vital role in supporting their use of e-health [6].

Based on the findings in this study, the research team has decided on some changes in the support program. Within the program it was possible to register blood pressure daily. Some patients did not notice that it was optional and thereby perceived it as burdensome. Therefore, in a revised version of the support program, it is possible to adapt intervals for registration of blood pressure based on the individual needs of each patient. Some patients had not looked through all the modules in the program, and therefore a pamphlet presenting the support program and the features within the program has been developed. Although the program content is presented in the first module, providing instructions through multiple media formats could further enhance participants’ comprehension and engagement with the program.

The findings also prompted revisions in certain aspects of the planned RCT. The nurses had concerns regarding the timing of when to introduce the support program, since patients might be overwhelmed with all the information when they are newly diagnosed and perhaps meeting the nurse for the first time. In addition, patients included in this study that have had heart failure for a long time still were active in using the support program. Therefore, the inclusion criteria for the upcoming RCT have been revised, as we decided to not only include patients with newly diagnosed heart failure. Another key insight gained from this study is that since many of the patients accessed only a few of the modules, they might need reminders and additional support from their heart failure nurses to use the support program. In the interview the nurses discussed that they could facilitate the patients use of the support program, and this is also in line with findings form [6]. Therefore, the study protocol of the RCT now includes that the nurses will send reminders to patients to use the support program.

### Study strengths and limitations

This study has some limitations. First, the sample size was small, which is appropriate for a feasibility study aimed at assessing the practicality of an intervention before larger trial [11]. As only four nurses participated, a focus group interview was chosen to facilitate discussion and capture diverse perspectives [22]. Second, not all patients completed all modules, which may have influenced their responses. A more controlled approach—such as reviewing all modules with a researcher—could have reduced this limitation. However, feasibility studies are designed to explore how interventions function in real-world settings and to identify necessary adaptations before further evaluation [11, 14]. Third, for some patients, several weeks had passed since their last login, posing a risk of recall bias, though this was intentional to assess longer-term use.

However, data collection and analysis followed a structured procedure guided by feasibility study frameworks and the program was developed in co-design with end-users. The study did not evaluate efficacy; further research is therefore warranted.

### Recommendations for further research

Future research should explore the long-term effects and clinical outcomes of e-health interventions such as the *“Living with Heart Failure”* programme, including their impact on self-care behaviour, symptom management, and quality of life. Larger, controlled studies are needed to evaluate cost-effectiveness and scalability across diverse healthcare settings.

Further investigation into factors influencing engagement and sustained use among patients and healthcare professionals would provide valuable insights for implementation strategies. Research should also address how digital competence and attitudes towards technology shape adoption and integration in clinical practice.

Finally, incorporating participatory and co-design methodologies in future studies may enhance the relevance, acceptability, and sustainability of e-health interventions in heart failure care.

### Implications for policy and practice

Implementing e-health interventions such as the *“Living with Heart Failure”* programme requires both organisational and individual readiness. Healthcare organisations should allocate sufficient time and resources to enable nurses to introduce and demonstrate the programme effectively to patients. Integration within existing national platforms such as 1177 is advantageous but necessitates improved interface design and clearer navigation to enhance accessibility for users.

In clinical practice, heart failure nurses play a central role in promoting patient engagement and sustained use. Structured onboarding, regular reminders, and clear communication through the platform can strengthen adherence. To support successful implementation, healthcare professionals require ongoing digital competence development and dedicated time during patient consultations.

As the support program provides the possibility for patients to register blood pressure and body weight and can alert the nurses when any registration is outside of acceptable range, it also allows heart failure nurses an opportunity to better monitor the patients. The program can increase patients’ self-management by providing relevant, easy-to-understand information. Patients can also take a knowledge-test with instant feedback to evaluate their knowledge and understanding of self-care recommendations.

At a policy level, co-design approaches, user feedback, and continuous evaluation should be prioritised when scaling up digital interventions to ensure usability, equity, and effective integration into routine heart failure care. These findings underline the feasibility and practical relevance of incorporating digital support into existing heart failure care pathways.

## Conclusion

In conclusion, the support program was deemed feasible by both patients and nurses in terms of acceptability, demand, and practicality. The nurses identified time as a key barrier for implementing the program. The feasibility study highlighted the need for certain modifications of the features in the program and that the use of the support program is not limited to patients newly diagnosed with heart failure. Even patients who have been living with heart failure for an extended period may benefit from the support program.

## Supplementary Information

Below is the link to the electronic supplementary material.

Supplementary Material 1

## Data Availability

The data thar support the findings of this study are available from the corresponding author upon reasonable request.
