# Supplementary material for: ”Living with heart failure” –patients’ and nurses’ experience using an online support program via a national e-health platform – a feasibility study
Source: BMC Nurs. 2026 Feb 4;25:202. doi: 10.1186/s12912-026-04370-z (PMC12958725; doi:10.1186/s12912-026-04370-z)
Supplement: Supplementary file 1 — Supplementary Material 1 [file 12912_2026_4370_MOESM1_ESM.docx]

# Interview Guide

## Introductory Questions

1. What channels do you usually use to contact healthcare services?
2. Do you usually use the 1177 platform to communicate with healthcare services?
3. Are you accustomed to using a computer or smartphone?
4. Have you logged in to/used the support program?

## For Participants Who Did Not Log In to the Program

We would like to understand the reasons why you did not log in to the program and explore what we could do in a future study to make it easier and ensure that all patients are able to access the program.

1. Did you at any point try to log in to the support program?
2. What is the reason that you have not used the program?
3. What support or assistance would you have needed in order to start using the program?
4. Do you think a support program could be beneficial for people with heart failure?
5. What support or assistance do you think other people with heart failure might need?
6. Finally, is there anything else you would like to share?

## For Participants Who Have Used the Program

With the following questions, we aim to explore your experience of using the support program. The purpose is to determine whether the program is user-friendly or whether any adjustments are needed.

1. What was your first impression of the program?
2. Starting from the beginning, what was it like for you when you first tried to log in to the program?
   - Was it easy? Was it difficult?
   - Was the written information helpful?
   - Did you need help logging in to the program? If so, who helped you? Was the written information helpful?
   - Did you use the program on a computer, phone, or tablet?
   - Did you receive the support you needed?
   - If not, what kind of support would you have needed?
3. How did you experience navigating the program? (Was it easy or difficult to understand how to move around and find things in the program?)
4. How did you experience the appearance of the program? (For example, design, text size, images, colors.)
5. Have you used the support program together with a family member or someone close to you?

## Functions

Now we will ask a few questions about your experience using the different functions available in the program.

1. Did you use the option to enter your weight or blood pressure? How did you experience that? Did entering, for example, your weight add anything of value for you?
2. Did you answer the quiz questions? How did you experience that?
3. Did you use the option to send messages to the nurse? How did you experience that?
4. Did you use the option to fill in the timeline (e.g., scheduled visits or planned blood tests)? How did you experience that?

## Content

Now we will ask some questions about your opinions on the content of the program.

1. Was there any part of the program that was particularly important to you?

- Did you learn anything from using the program?

1. How did you experience the overall scope of the program?

- Is there anything you would have liked to know more about, or is there any content you felt was missing?
- Did you find the program too extensive?

## What Can We Improve for the Next Study?

These questions will help us plan for the upcoming study.

1. For how long do you think it is useful to have access to the program?
2. If you had the opportunity, would you want continued access to the program after this study is completed?
3. Would you recommend the support program to others with heart failure? Why?
4. Finally, is there anything else you would like to share?
